# Less Is More in Biosignal Analysis: Compressed Data Could Open the Door to Faster and Better Diagnosis

**DOI:** 10.3390/diseases6010018

**Published:** 2018-02-24

**Authors:** Mohamed Elgendi

**Affiliations:** 1 Department of Obstetrics & Gynecology, University of British Columbia and BC Children’s & Women’s Hospital, Vancouver, BC V6H 3N1, Canada; mohamed.elgendi@cw.bc.ca; Tel.: +604-600-4139; 2 School of Electrical and Computer Engineering, University of British Columbia, Vancouver, BC V6T 1Z4, Canada

**Keywords:** signal processing, global health, digital health, mobile health, big data, algorithm design, bit-rate reduction

## Abstract

In the digital medicine field, biosignals, such as those of an electrocardiogram (ECG), are collected regularly for screening and diagnosis, and there continues to be an increasingly substantial shift towards collecting long-term ECG signals for remote monitoring, e.g., in smart homes. ECG signal collection is quite simple and only requires the use of inexpensive sensors, an active Internet connection, and a mobile device that acts as the medium between the sensors and the Internet (e.g., a mobile phone or laptop). Despite the ease and convenience of remote ECG data collection and transmission, the amount of time and energy required for the related remote computational processes remains a major limitation. This short note discusses a biosignal approach that uses fewer biomedical data for screening and diagnosis that is, compared to current data collection methods, equally, if not more, efficient.

## 1. Introduction

Advances in healthcare tend to focus on developing complex algorithms: the programs behind the flashy apps that can track people’s health do not thoughtfully consider the transmission and analysis of the substantial amounts of collected data [1]. Meanwhile, efforts to introduce sweeping changes to the complex algorithms to make them more suitable for big data have been ignored [2]. Moreover, algorithms for big data collected in medicine, especially in cardiovascular care, are still in the early stages of development and evaluation and lack evidence of improving quality of care and patient outcomes [3].

More and more, clinicians are starting to demand the long-term collection of ECG signals and the online monitoring of these signals to better understand the progression and life cycle of certain diseases [4]. This data can help practitioners provide better care and treatment for their patients [5]. With this demand comes an increased pressure on biomedical engineers to develop more complex algorithms that collect larger data sets that are complete and do not allow for the removal of any data points [6]. This can be problematic since larger data sets require more complex computational resources to collect, analyze, and transmit the data and require large data centers that are resource-exhaustive.

## 2. Case Description

A recent approach [7,8] published last year through the University of British Columbia (Canada) used a different method; the method described a novel way of transmitting ECG data so that it requires less space (memory) and less time to process and transmit the data, all while still providing an accurate analysis that is comparable to previous approaches that use full data sets collected in conventional ways. The proposed approach speeds up computational performance and can be used for any biosignal, such as that of a photoplethysmogram or an ECG. In fact, the proposed approach is generic and applicable to any time-series big data application. More importantly, the new approach uses fewer resources and transmits compressed information (i.e., less information from the ECG signal) while protecting the data’s integrity and preserving the essential data components. Preserving data integrity is necessary, as it ensures that disease diagnosis can be carried out accurately and properly.

## 3. Discussion

This proposed compression approach is applied to one-dimensional data (e.g., ECG signals) that are typically collected with higher sampling frequencies, such as 1000 Hz [9] (i.e., in order to transmit a one-second snapshot of the patient’s data to a physician, 1000 data points are required). This common collection approach overwhelms the device that is used to collect the ECG signal, the servers on the Internet, and the receiving device the clinician uses to read the data. By compressing the ECG data (i.e., removing redundant components), the resource burden is lessened, thereby increasing the collection, transmission, and analysis processes. The essential information of the ECG signal (i.e., polarization and depolarization) is kept by using an interpolation process, therefore preserving the necessary features of the biosignal.

Interestingly, during the validation process of the compression approach, the mathematical analysis demonstrates that the main features of the biosignal are amplified before compression, making them more prominent and informative. Consequently, when the compression step is executed after the amplification step, the main ECG features still hold strong producing higher heartbeat detection accuracy. It’s an intriguing, thought-provoking idea, detecting heartbeats from compressed ECG is better than using original (uncompressed) ECG signal. The compression process can be viewed as a filter, in that it extracts redundant data and leaves a purer presentation of the ECG features.

Of course, when we speak about algorithms, the implementation of the algorithm affects both the software and hardware. The compression algorithms are the software, while the hardware must allow the software to advance. This is a challenge that needs to be addressed as well in order to take advantage of new machine learning techniques. For example, currently, the data-storage units inside computers are separate from data-processing units. This creates a bottleneck in performance, time, and power. For decades, advances in computing have been driven by scaling down the size of the components, guided by Gordon Moore’s prediction that the number of transistors on a chip will roughly double every two years; however, this prediction did not consider the processing power, especially of mobile phones (or battery-driven devices). In the upcoming decades, the hardware implementation of biosignal analysis for collecting big data needs to change accordingly to enable fast and reliable decision making.

## 4. Conclusions

Currently, the proposed compression method has been tested on one-dimensional time-series data; however, it can be applied to two-dimensional data (e.g., MRI pictures) with some modifications. The principle of this compression method can work on any biosignal data or time-series data that contains periodic (repeated) features, and it has mass application potential for big data. More importantly, the compression biosignal approach demonstrates that using fewer biomedical data is as efficient as, and even better than, current data collection, transmission, and analysis methods used in remote monitoring, screening, and diagnosis. Big data thinking has commonly been that the more data you have, the better the understanding and insight you will have into the area of interest. Contrary to this belief, fewer data can be as informative, if not more informative, when paired with efficient compression and analysis algorithms.
